# Mapping where and when necroptotic cell death occurs in disease

**DOI:** 10.1038/s41418-024-01318-1

**Published:** 2024-06-04

**Authors:** Andre L. Samson, James M. Murphy

**Affiliations:** 1 Walter and Eliza Hall Institute of Medical Research, Parkville, VIC Australia; 2 University of Melbourne, Parkville, VIC Australia; 3 Drug Discovery Biology, Monash Institute of Pharmaceutical Sciences, Parkville, VIC Australia

**Keywords:** Cell death and immune response, Cell biology

## Abstract

A series of recent studies have established where the necroptosis machinery — RIPK1, RIPK3, MLKL, ZBP1 and Caspase-8 — occur and are activated in mouse and human tissues, and how ZBP1 splicing might regulate necroptosis. These studies highlight the importance of mapping necroptosis spatially to understand how pathway dysregulation can lead to disease.

Necroptosis is a form of programmed cell death characterized by the overt loss of membrane integrity, the release of cellular constituents and the paracrine promotion of inflammation. Necroptosis was first discovered as a caspase-independent response to tumor necrosis factor. Today, it is known that a wide variety of immune and inflammatory stimuli can trigger necroptotic cell death (Fig. 1A). These pro-necroptotic signals instigate the oligomerisation of RIP Homotypic Interacting Motif (RHIM)-containing proteins. Receptor-Interacting serine/threonine Protein Kinase 1 (RIPK1), RIPK3, Z-nucleic acid binding protein (ZBP1) and TIR domain–containing adapter–inducing interferon-β (TRIF) are the most studied RHIM-containing proteins in mammals (Fig. 1A). These RHIM-containing scaffolds can then trigger RIPK3 oligomerisation and auto-phosphorylation under specific cellular contexts, such as when Caspase activity is low. Phospho-activated RIPK3 then transphosphorylates Mixed Lineage Kinase domain-Like (MLKL), which in turn promotes a series of conformational changes in MLKL that culminate in it disrupting membranes to cause lytic cell death (Fig. 1A).

**Fig. 1:**
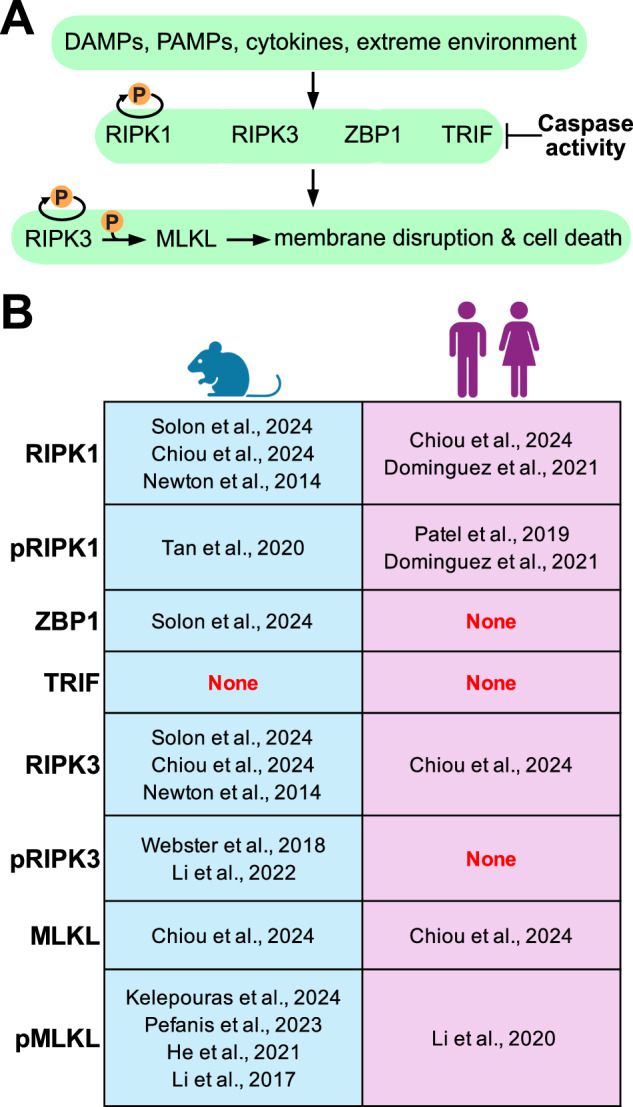
The necroptosis pathway and its immunohistochemical detection. **A** Schematic of the stimuli, RHIM-domain containing mediators, and terminal effectors that comprise the necroptosis pathway. **B** Papers that provide detailed chromogenic immunohistochemical methods to detect the stipulated targets.

To uncover these fundamental steps in the necroptotic cascade, researchers have heavily relied on measuring phenotypic changes produced by genetic knock-out/-in strategies (e.g., *Mlkl*^*−/−*^ mice) or by small molecule inhibitors (e.g., Nec-1s). Often, these phenotypic changes are correlated with differences in necroptotic signaling as measured by western blot. While these collective approaches allow concrete conclusions to be drawn about the roles of necroptosis, they provide little insight into the sites of necroptotic signaling in cells and tissues. Indeed, as with the use of PD-L1 immunohistochemistry to stratify patients for immune-checkpoint therapy, spatially-resolved techniques will likely be needed to guide the translation of necroptosis-targeted therapies. Accordingly, studies are increasingly using spatial approaches to study necroptotic signaling [1–17]. To promote these efforts, Fig. 1B provides a list of studies with detailed chromogenic immunohistochemical methods to detect necroptotic pathway expression and activation in tissues. The current inability to detect human ZBP1, TRIF and pRIPK3 using in situ techniques is highlighted (Fig. 1B).

Three new studies have expanded the field’s spatial capabilities [18–20]. Kelepouras et al. optimized an immunohistochemical method to detect MLKL phosphorylated at serine 345 (pMLKL), a widely used marker of necroptotic signaling in mice [19]. Importantly, the specificity of this immunoassay was validated using two negative controls: 1) *Ripk3*^*−/−*^or *Mlkl*^−/−^ mice, and 2) the dephosphorylation of MLKL in tissue sections using λ phosphatase. This immunohistochemical method was used to pinpoint MLKL activation in two established models of necroptosis: 1) *Casp8*^E-KO^ mice which lack expression of Caspase-8 in keratinocytes, and 2) *Sharpin*^cdpm/cdpm^ mice with excess inflammation owing to dysregulated linear polyubiquitination. In *Casp8*^E-KO^ mice, necroptosis preferentially occurred in epidermal keratinocytes, whereas apoptosis arose in the dermis. In *Sharpin*^cdpm/cdpm^ mice, necroptosis was noted in the spleen, whereas apoptosis was more prevalent in the skin. It is interesting that necroptosis and apoptosis affected distinct cell populations in these models, as it suggests that these subroutines of cell death are under strict spatial control. Kelepouras et al. also showed that intestinal apoptosis, rather than necroptosis, arises in mice after systemic TNF administration. Others have performed similar studies using immunohistochemistry to detect pRIPK3 and pMLKL [3, 6, 21] and reached different conclusions about the induction of necroptosis after systemic TNF challenge. These differing conclusions highlight the need for further standardization of immunohistochemical procedures to study necroptosis.

To this end, two new papers have provided automated immunohistochemistry protocols for the necroptotic pathway: Chiou et al. [18] and Solon et al. [20]. We profiled the expression of Caspase-8, RIPK1, RIPK3 and MLKL in human and mouse tissues [18]. We found that the necroptotic pathway is basally expressed by immune cells and barrier cells, whereas expression of the pathway was low or absent in many long-lived populations, such as post-mitotic neurons in the adult mouse. We also showed that RIPK3 and MLKL are rapidly and locally upregulated during immune and inflammatory challenge. Together, these findings support the idea that necroptosis is an innate anti-pathogen defensive measure and that the necroptotic pathway is tightly controlled at the spatial level. As an alternative to detecting pRIPK3 or pMLKL, we proposed that intracellular accumulations of the necroptotic pathway—which likely represent the RHIM-mediated oligomeric structure known as the necrosome—can be used to pinpoint necroptotic signaling in situ. Finally, we used automated techniques to identify the sites of necroptotic signaling in patients with inflammatory bowel disease. Reminiscent of the observations of Kelepouras et al., a dichotomy between apoptosis and necroptosis was noted in patients with inflammatory bowel disease.

An independent study by Solon et al. also used automated immunohistochemistry to provide an atlas of the necroptotic pathway in mouse tissues. Specifically, they surveyed the expression patterns of ZBP1, RIPK1, and RIPK3 protein, together with *Mlkl* transcript levels, across ~27 mouse tissues [20]. This comprehensive overview underscores the conclusion that the necroptotic pathway is constitutively expressed by many immune subsets and by barrier-forming cells such as the endothelium and intestinal epithelia. Solon and colleagues then showed that ZBP1 expression, or ZBP1^+^ cell infiltration, increases in tissues prior to the ZBP1-dependent death of *Casp8*^*−/−*^*Tnfr1*^*−/−*^embryonic mice. This finding points to the existence of a sterile TNF-independent trigger for ZBP1-mediated signaling, adding weight to the notion that ZBP1 serves roles beyond the sensing of pathogen-derived nucleic acids.

Adding complexity to necroptotic signaling is the many isoforms and proteolytic fragments in the necroptotic pathway (e.g., UniProt lists 7 isoforms of human ZBP1). Except for caspase-targeted protocols, current immunostainings ignore the likelihood of functionally important isoforms and cleavage events. The study by Koerner *et al*. brings this issue into sharp focus [22]. They identified a truncated form of mouse ZBP1, which when expressed in cells and tissues triggered both necroptosis and Caspase-8-dependent apoptosis. Where this truncated ZBP1 isoform localizes in cells is a fascinating question, especially as it lacks two RHIM domains. Application of suitable immunostaining protocols to tissues from mice expressing this truncated ZBP1 isoform may illuminate whether apoptosis and necroptosis are still spatially segregated despite a common cell death trigger. The precise functions of the human ZBP1 isoforms, and how they differ from those expressed by mice, await illumination as suitable reagents for spatial monitoring emerge.

While enormous progress has been made, in addition to the distinct roles of effector isoforms highlighted by Koerner et al. [22], an important challenge remains that the necroptotic pathways in mice and humans differ markedly (e.g., the average identity for the proteins in Fig. 1A is only 60%), necessitating that most of the immunohistochemistry protocols in Fig. 1B are species-restricted. The recent advances of Kelepouras et al., Chiou et al., and Solon and colleagues, as well as those cited in Fig. 1B, highlight the important knowledge that can be gained by spatially mapping where necroptosis occurs in diseased tissues. The development of selective and sensitive immunohistological antibodies has been instrumental in enabling detection of necroptotic events in tissues, which we are optimistic will continue as knowledge of the pathway in disease advances.

## References

[1] Dominguez S, Varfolomeev E, Brendza R, Stark K, Tea J, Imperio J (2021). Genetic inactivation of RIP1 kinase does not ameliorate disease in a mouse model of ALS. Cell Death Differ.

[2] Chen X, Zhu R, Zhong J, Ying Y, Wang W, Cao Y (2022). Mosaic composition of RIP1-RIP3 signalling hub and its role in regulating cell death. Nat Cell Biol.

[3] He P, Ai T, Yang ZH, Wu J, Han J (2021). Detection of necroptosis by phospho-MLKL immunohistochemical labeling. STAR Protoc.

[4] Li D, Ai Y, Guo J, Dong B, Li L, Cai G (2020). Casein kinase 1G2 suppresses necroptosis-promoted testis aging by inhibiting receptor-interacting kinase 3. Elife.

[5] Li D, Meng L, Xu T, Su Y, Liu X, Zhang Z (2017). RIPK1-RIPK3-MLKL-dependent necrosis promotes the aging of mouse male reproductive system. Elife.

[6] Li L, Huang K, Ruan C, Han J, Zhang Y (2022). Immunostaining of phospho-RIPK3 in L929 cells, murine yolk sacs, ceca, and small intestines. STAR Protoc.

[7] Murai S, Takakura K, Sumiyama K, Moriwaki K, Terai K, Komazawa-Sakon S (2022). Generation of transgenic mice expressing a FRET biosensor, SMART, that responds to necroptosis. Commun Biol.

[8] Nemegeer J, Lemeire K, Vandenabeele P, Maelfait J. Tyramide signal amplification for the immunofluorescent staining of ZBP1-dependent phosphorylation of RIPK3 and MLKL after HSV-1 infection in human cells. J Vis Exp. 2022. 10.3791/64332. 10.3791/64332 36342164

[9] Newton K, Dugger DL, Wickliffe KE, Kapoor N, de Almagro MC, Vucic D (2014). Activity of protein kinase RIPK3 determines whether cells die by necroptosis or apoptosis. Science.

[10] Patel S, Webster JD, Varfolomeev E, Kwon YC, Cheng JH, Zhang J (2020). RIP1 inhibition blocks inflammatory diseases but not tumor growth or metastases. Cell Death Differ.

[11] Pefanis A, Bongoni AK, McRae JL, Salvaris EJ, Fisicaro N, Murphy JM (2023). Dynamics of necroptosis in kidney ischemia-reperfusion injury. Front Immunol.

[12] Rodriguez DA, Quarato G, Liedmann S, Tummers B, Zhang T, Guy C (2022). Caspase-8 and FADD prevent spontaneous ZBP1 expression and necroptosis. Proc Natl Acad Sci USA.

[13] Samson AL, Fitzgibbon C, Patel KM, Hildebrand JM, Whitehead LW, Rimes JS (2021). A toolbox for imaging RIPK1, RIPK3, and MLKL in mouse and human cells. Cell Death Differ.

[14] Samson AL, Zhang Y, Geoghegan ND, Gavin XJ, Davies KA, Mlodzianoski MJ (2020). MLKL trafficking and accumulation at the plasma membrane control the kinetics and threshold for necroptosis. Nat Commun.

[15] Tan S, Zhao J, Sun Z, Cao S, Niu K, Zhong Y (2020). Hepatocyte-specific TAK1 deficiency drives RIPK1 kinase-dependent inflammation to promote liver fibrosis and hepatocellular carcinoma. Proc Natl Acad Sci USA.

[16] Webster JD, Solon M, Haller S, Newton K (2018). Detection of necroptosis by phospho-RIPK3 immunohistochemical labeling. Methods Mol Biol.

[17] Zhang T, Yin C, Boyd DF, Quarato G, Ingram JP, Shubina M (2020). Influenza virus Z-RNAs induce ZBP1-mediated necroptosis. Cell.

[18] Chiou S, Al-Ani AH, Pan Y, Patel KM, Kong IY, Whitehead LW, et al. An immunohistochemical atlas of necroptotic pathway expression. EMBO Mol Med. 2024. 10.1038/s44321-024-00074-6. 10.1038/s44321-024-00074-6 PMC11250867 38750308

[19] Kelepouras K. The importance of murine phospho-MLKL-S345 in situ detection for necroptosis assessment in vivo. Cell Death Differ. 2024. 10.1038/s41418-024-01313-6. 10.1038/s41418-024-01313-6 PMC11239901 38783091

[20] Solon M, Ge N, Hambro S, Haller S, Jiang J, Baca M (2024). ZBP1 and TRIF trigger lethal necroptosis in mice lacking caspase-8 and TNFR1. Cell Death Differ.

[21] Deng B, Yang D, Wu H, Wang L, Wu R, Zhu H (2022). Ketamine inhibits TNF-alpha-induced cecal damage by enhancing RIP1 ubiquitination to attenuate lethal SIRS. Cell Death Discov.

[22] Koerner L, Wachsmuth L, Kumari S, Schwarzer R, Wagner T, Jiao H. ZBP1 causes inflammation by inducing RIPK3-mediated necroptosis and RIPK1 kinase activity-independent apoptosis. Cell Death Differ. 2024. 10.1038/s41418-024-01321-6. 10.1038/s41418-024-01321-6 PMC11239871 38849574

